# COVID-19 in Canada: A self-assessment and review of preparedness and response

**DOI:** 10.7189/jogh.10.0203104

**Published:** 2020-12

**Authors:** Alice Yu, Sophia Prasad, Adebisi Akande, Andreea Murariu, Serena Yuan, Sylvia Kathirkamanathan, Myles Ma, Sarah Ladha

**Affiliations:** 1 Faculty of Science, University of Western Ontario, London, Ontario, Canada; 2 Department of Biological Sciences, University of Toronto, Scarborough, Ontario, Canada; 3 Department of Biological Sciences, University of Toronto, Scarborough, Ontario, Canada; 4 Richmond Hill High School, Richmond Hill, Ontario, Canada; 5 Faculty of Arts and Science, University of Toronto, St. George, Ontario, Canada; 6 Faculty of Arts and Science, Queen’s University, Kingston, Ontario, Canada; 7 Faculty of Science, University of Waterloo, Waterloo, Ontario, Canada; 8 College of Health, Community and Policy, University of Texas at San Antonio, San Antonio, Texas, USA

## COVID-19 PANDEMIC

The global pandemic caused by the novel coronavirus, COVID-19, has overturned the stability of public health systems and economies in countries all over the world. Much about the virus itself remains unknown; the outbreak began in Wuhan, China, but its origins are still largely speculative. The predominant belief is that the virus was transferred to humans from bats, as a novel virus with 88% similarity with COVID-19 [1]. The virus spread extremely quickly in comparison to similar diseases such as SARS and no consensus has been reached for strategies of containment [2]. As such, despite World Health Organization guidelines, countries have been implementing independent responses that have broadly failed to contain the pandemic [2]. These policies typically include quarantines, restricting travel, limiting public gatherings, and expanding public health programs to accommodate an increased number of sick patients and testing requirements [2]. With no end in sight, it is imperative for Canada to consider a wider variety of tactics to combat the virus altogether in addition to learning from outcomes in other countries. Although researchers have produced many topic-focused findings concerning individual policies and scientific recommendations, there is a lack of extensive reviews covering a wide range of data collected from multiple countries on multiple pandemic response strategies. Such comprehensive review provides a birds-eye, analytical approach to coordinating multiple sectors of governance within the realm of public health, from mental health to elderly care homes.

## REVIEW OF CANADA IN GLOBAL CONTEXT

In March of 2020, Canada faced a surge of unnecessary hospital visits in Toronto, which were largely due to a lack of personal protective equipment, diagnostic testing, and proper protocol for community health care providers [3]. While Canada has since adopted more widespread testing [4] and implemented measures that were effective, these responses were delayed, and the country is struggling to control the pandemic. As of June 24, 2020, Canada's case-fatality rate (8.29%) was higher than that of both the global average (5.17%) and the United States (5.17%), which holds the record for the highest number of COVID-19 deaths [5]. Asian countries initially suffered some of the worst effects of COVID-19. These countries have comparatively optimistic case fatality rates despite their proximity to the first identified case of the virus, larger and denser populations, and less prepared health care systems as determined by the Global Health Security Index (GHS Index) [6]. One notable example is Singapore with a case-fatality rate as low as 0.06% [7].

**Figure:**
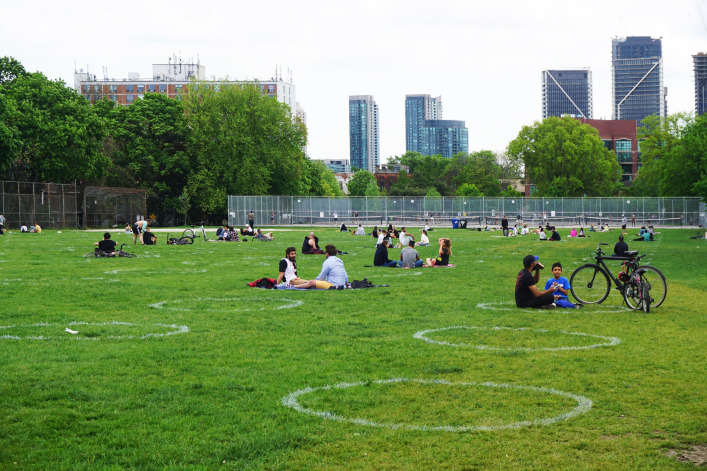
Photo: Locals use social distancing circles in the Trinity-Bellwoods park of Toronto on a weekend during coronavirus pandemic in Ontario, Canada (purchased with a Standard Use License from: https://www.istockphoto.com/photo/trinity-bellwoods-park-of-toronto-during-pandemic-gm1245043358-363037303). Credit: By lavendertime on iStock.

As COVID-19 became a global pandemic, each country adopted unique and specific policies to keep citizens safe and maintain functioning economies. Non-pharmaceutical measures (testing, isolation, quarantine, contact tracing, and physical distancing) were implemented to suppress the pandemic prior to the development of a vaccine [8]. In this study, Canada was analyzed alongside six other countries: the United States, Spain, Taiwan, Singapore, China, and South Korea under different topics. A basis for comparison was established in that all countries were ranked high or upper-middle income according to the GHS Index, and all were ranked in the top 12.3% of countries in the overall GHS Index [6]. All countries, except for China, were in the top 35 out of 195 countries ranked by Gross Domestic Product (GDP) per capita, as measured by Purchasing Power Parity (PPP) [9]. China was included in the study given that currently, it is assumed to be the origin of COVID-19, and many studies looked to their first-hand experience with the novel coronavirus. These shared national characteristics showed that these countries had reasonably similar struggles and public health systems in the context of the pandemic. Thus, recommendations drawn from these countries are relevant to the future improvement of Canada.

It is hypothesized that Canada’s passive approach, relative to other nations, resulted in poorer outcomes for its health care system, specifically, higher number of cases, COVID-19 related deaths, and lower testing rates. It is also predicted that Canada had the same issue with protecting those in nursing homes as those in the United States. The primary objective of this article is to conduct a self-assessment of Canada’s response to the pandemic through topics of health care operations, pandemic policies, testing policies, lessons from the SARS epidemic, early responses, public communications, mental health and school closures. This study will also incorporate and compare some international responses in the self-assessment.

We conducted a review regarding the preparedness and response of Canada during the COVID-19 pandemic. Articles were collected from the following research domains: PubMed, EMBASE, Google Scholar and MEDLINE (Ovid). Article retrieval and data extraction were conducted in two areas: the response and preparedness of six countries (United States (US), Spain, Taiwan, Singapore, China, and South Korea) as well as the response and preparedness of the Canadian health care system. We searched for all journal articles using the following terms as keywords: “COVID-19, 2019-nCOV, SARS, testing, preparedness, response, SARS-CoV-2, and coronavirus.” Moreover, news articles were retrieved using Google News. Keywords used include “COVID-19, 2019-nCOV, preparedness, response, SARS, testing, SARS-CoV-2 and coronavirus.

Initial screenings to determine eligibility of potential articles for inclusion primarily involved analysis of article titles and abstracts. Articles that were deemed suitable for inclusion underwent a full-text review. In this stage, we aimed to find articles that discussed the preparedness and response of the Canadian government as well as those of the United States (US), Spain, Taiwan, Singapore, China, and South Korea during the pandemic. Given the various metrics that countries used to measure preparedness and responsiveness to the pandemic, we aimed to include articles that analyzed and measured the following: testing policy, contract tracing surveillance, containment measures, lockdown procedures, public communication, hospital preparedness and management, and management in long-term care facilities. Only one unpublished study conducted in Thailand and one study conducted in Italy were included in the Mental Health Section as they provided unique findings and insight into the psychological effects of the pandemic. A full list of eligibility criteria is shown in **Table 1**.

**Table 1:** Eligibility criteria for article selection

Inclusion	Exclusion
Peer-reviewed and published works	Non-English peer-reviewed studies, news articles and government press releases
News articles	
Government press releases	
Peer-reviewed studies, articles and press releases that highlight any aspect of a country’s preparedness and response to the pandemic	Peer-reviewed studies, data, news articles and government press releases that are not from the following countries: Canada, United States (US), Spain, Taiwan, Singapore, China and South Korea
Peer- reviewed studies, news articles and government press releases from Canada	
Peer-reviewed studies, news articles and government press releases from the following countries: United States of Amerika, Spain, Taiwan, Singapore, China, and South Korea	

The following descriptive information was extracted from the included articles: title, abstract, country, date of publication and types of preparedness and/or responsiveness highlighted in the article. Where possible, data was further extracted to record quantitative measures such as number of cases, case-mortality rate, number of tests and hospitalizations rates. Moreover, data was also extracted to record timelines of containment measures, lockdown procedures, case detection and testing policies, and logistical changes in hospitals and long-term care facilities. Articles that were deemed eligible for full-text review were retrieved and underwent data extraction, which was performed by all investigators using a shared online document.

## EFFECTIVE MEASURES

### Healthcare Operations

Canadian hospitals have efficiently adapted to the numerous stresses brought by the pandemic. For instance, Canadian hospitals have quickly restructured their emergency departments (ED) in order to facilitate an increase in health care delivery. A hospital in North York was able to completely reinvent its ED and continue operations within 33 hours [10]. Likewise, rural hospitals restructured patient flow protocols and built additional infrastructure for drastic patient increase that the smaller institutions were not equipped to accommodate in normal circumstances [11].

**Figure 1:**
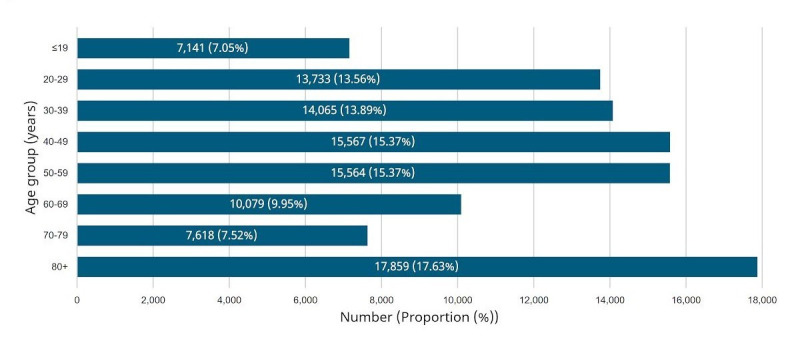
Photo: Graphic representation of age distribution of COVID-19 cases (n = 101 626) in Canada as of June 23, 2020 [12].

**Figure 2:**
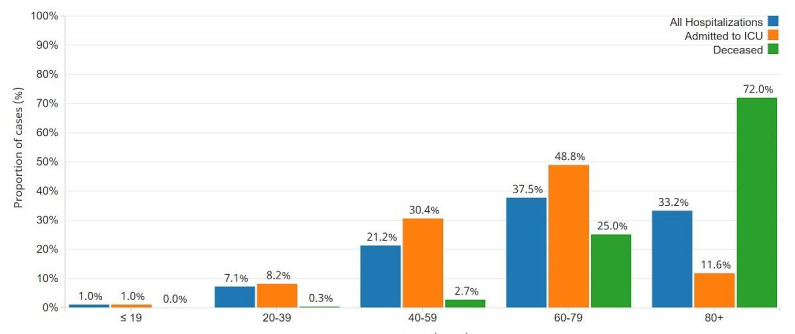
Photo: Graphic representation of age distribution of COVID-19 cases hospitalized, admitted to ICU and deceased in Canada as of June 23, 2020 [12].

Conversely, Long-Term Care (LTC) facilities haven’t had the same fortune when responding to the pandemic. The most notable amount of cases (17.63%) and deaths (72.0%) can be seen in the 80 year-old old + age group in Canada as seen in **Figure 1** [12]. This is explained by previous studies, which have shown that those above the age of 65 and with pre-existing comorbidities are at a higher risk of death [13,14]. However, there is also a considerably low percentage of admission to the ICU for this age group in addition to higher death rates as seen in **Figure 2** [12].

Moreover, this increased risk of cases and deaths within the 80 year-old old + demographic can be attributed to the numerous inadequacies that plague LTC facilities. Weak regulations and budgetary constraints have led to issues such as the lack of available rooms, poor working conditions and low quality of care for its residents [15]. As a result of these issues, LTC facilities have had to recruit help from hospitals in order to improve their quality of care. In one study, a hospital in Ontario was called in to intervene at a nursing home that saw 12 deaths and 89 infected residents as well as 49 infected staff members [16]. The partnering hospital was able to implement a four-phase plan (Table 2) that resulted in the stabilization of the outbreak [16].

**Table 2:** Summary of the four-phase plan*

**Engagement, relationship and trust-building** – Involved listening to the LTC about the situation; working collaboratively to problem solve
**Environmental scan, team-building and immediate response (first 72 hours)** – Identified immediate needs such as clinical care, testing etc.; team was constructed to execute emergency response; 15 patients sent to acute care hospital
**Early phase response (next 7 days)** – Mortality was at its peak; infrastructure built for palliative care, virtual care; residents were triaged and health records were collected; nursing home staff were trained
**Stabilization and transition phase (day 10 to present)** – Outbreak was stabilizing; phase focused on alleviating staffing shortages, optimizing the medical and psychiatric care of residents, and preparing the home for a transition back to more autonomous clinical care and management

*Adapted from [16].

### Government coordination of responses

A flaw in the global response to COVID-19 was poor government coordination. This was demonstrated within the geographic distribution of cases in the United States. A study had shown that the majority of cases in the USA were concentrated within certain counties and states, with ten states accounting for approximately 70% of cases and 75% of deaths. The investigators concluded that since certain regions are disproportionately affected by the pandemic, it is imperative that the government avoid blanket policies and create geographically specific policies [17].

In contrast, various regions in Spain enacted different policies at different times. This led to worse results than the USA, as seen by a simple comparison of case fatality rates: 3.9% in the USA and 11.0% in Spain [7]. In Spain, Basque Country declared COVID-19 a public health emergency before any other region, and Catalonia asked for a complete shutdown of the region including restricting access to ports [18]. Madrid, La Rioja, and Vitoria banned gatherings of over 1000 people and implemented social distancing measures [18]. Although Spain’s health care system had initially coped well with the virus during the first few weeks, the country’s state quickly deteriorated, leading to an increase of approximately 1000 cases a day with the most affected regions being Basque Country, Catalonia, and Madrid [18]. Despite policies implemented at the regional level, along with other factors [18], the lack of communication between levels of government meant Spain’s response was staggered and ineffective. Based on the incidences in the USA and Spain, it is important to implement both general policies that are implemented throughout the entire country, like social distancing, as well as region-specific policies that encompass both virus hotspots and low-risk regions. To execute these policies, communication between different levels of government must be prioritized and regional policymakers need to make informed judgment calls on the timing and mandates of public health policies.

In comparison, Canada has had more success with the implementation of region-specific policies. The Canadian government enacted policies at different times depending on how much the virus spread within a given region. An example being that provinces closed their borders to interprovincial travel to prevent the spread of the virus from tourists into geopolitical regions with very few cases [19]. This allowed some provinces to reopen businesses, parks, and tourist attractions earlier than others as they were not impacted by the high infection rates in provinces such as Ontario and Quebec. School closures were also enacted at different dates at the community level, as well each school board was encouraged to devise individual reopening plans as appropriate [20]. Seeing these events, Canada has been able to create general and region-specific policies that allow different regions to respond effectively to the pandemic.

## AREAS OF IMPROVEMENT

### Early responses

**Figure 3:**
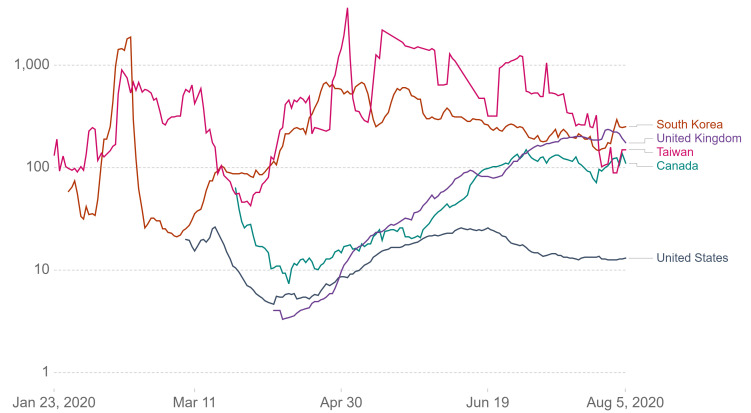
Photo: Graphic representation of tests conducted per new confirmed case from January 2 to August 5, 2020 in South Korea, United Kingdom, Taiwan and Canada. Tests are taken as rolling 7-day averages [5].

Canada’s unperturbed approach to the COVID-19 pandemic likely exacerbated its impacts more than necessary. In early simulations, the COVID-19 pandemic was shown to be highly responsive to early vigorous testing combined with close surveillance on human-to-human transmission [21], and intensified surveillance of imported cases can drastically reduce the probability of sustained transmission and the occurrence of an outbreak [22]. In addition, widespread testing before an epidemic outbreak can reduce the size of the entire epidemic and slight changes in the rate of testing can have sizable effects on the epidemic curve [23]. However, Canada faced difficulties in maintaining a high testing rate likely due to a shortage of laboratory supplies and logistic unpreparedness for the large testing capacity [24,25]. A “dip,” or decrease, in the testing rate before returning to the initial rate was seen in **Figure 3** [5] (*Testing Policies* section). This demonstrated a need for the stockpile of these essential supplies or a more readily-available supplier in preparation for epidemics.

Furthermore, Canada was relatively delayed in implementing lockdown procedures and facemask recommendations:

- Ontario announced lockdown procedures on March 17 [26], comparatively late to when WHO announced COVID-19 as a worldwide pandemic on March 11 [27].
- As early as February, numerous Asian officials recommended wearing face masks as a means of self-protection, whereas recommendations in Canada were given at a much later date [28]. The Chief Public Health Officer advised Canadians to wear a non-medical face mask on April 6 in locations where social distancing is difficult to maintain [29].
- On April 17, the Minister of Transport announced measures requiring all air passengers to have a non-medical mask or face coverings during travel and came into effect on April 20, 2020 [30].

These delayed actions suggested that Canada was not diligent in taking action during the early stages of the pandemic which may have been crucial in drastically reducing viral transmission. Further research using newly available data on COVID-19 can more accurately predict the effectiveness of early mass interventions. More definitive studies exploring the benefits of early enactment of lockdown and social distancing is also required in addition to mass testing and surveillance. Nevertheless, the possible advantages of early mass interventions are worth researching as it might be the key to reducing societal interactions and financial costs of arresting the economy.

In the evaluation on China, it was concluded that governments can adopt the Tri-Component Strategy (identifying sources of infection, the path of transmission, and vulnerable individuals) for any infectious disease without understanding its biology [21]. At the time of publication on March 2, 2020, this strategy was used by the Chinese government to successfully control the pandemic according to the same article. Combined with an existing Research and Development Blueprint from WHO [8] that entails rapid response activities, any passivity in times of a global pandemic is unjustifiable.

### Testing policies

As previously mentioned, testing is a crucial early response that Canada did not diligently utilise until months into the pandemic. Two main types of testing are currently available: viral DNA detection and serological tests. Serological tests are still being developed for time efficiency, and viral DNA detection using PCR testing is also relatively new and the sensitivity is not yet known with 0% chance to detect the virus on day 1 of infection and a 38% chance of a false-negative on day 5 of infection [31,32]. Despite its flaws, viral DNA detection is the primary testing method in Canada. Another area of concern is the passivity of testing. The first testing device was approved at the end of March 2020 and was slowly made available to the public [33]. Testing was also initially limited to individuals who had contact with confirmed cases or recommended by a physician after presenting with at least one symptom of COVID-19 [34].

**Figure 4:**
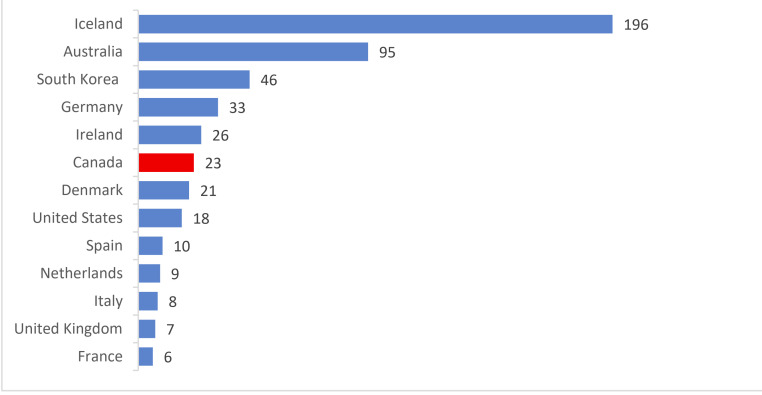
Positive COVID-19 tests per death in April 2020 [37].

**Figure 5:**
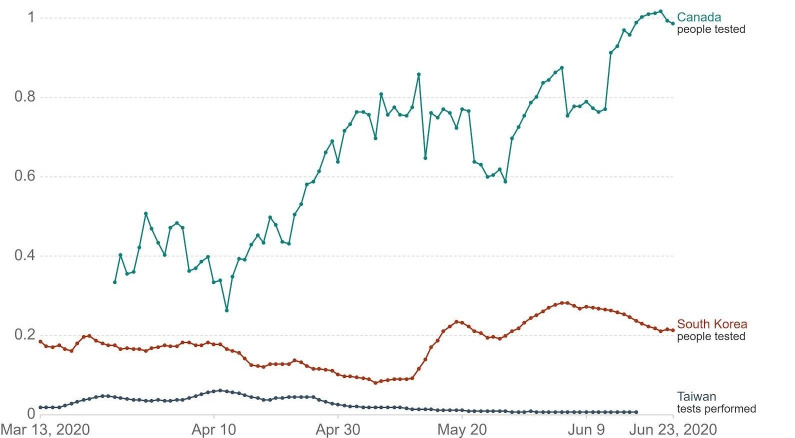
Photo: Graphic representation of daily COVID-19 tests per thousand people from March 13 to June 23, 2020 in Canada, South Korea, and Taiwan. Tests are taken as 7-day rolling averages [5].

South Korea was acknowledged as one of the successful examples in controlling the pandemic due to tactics of vigorous testing and case tracing [35]. Since the beginning of the pandemic, asymptomatic individuals could be tested beginning as early as late-January [36]. As a result, they observed 46 cases per death in April, where a higher case per death rate indicates effective use of testing to locate active cases. Meanwhile, Canada had 23 cases/death (**Figure 4**) [37]. Taiwan is another region recognized for their success in staging early interventions. Taiwan’s testing rate per confirmed case was on par with South Korea (**Figure 3**) [5]. However, the overall trend of testing rate per thousand people in Canada was significantly above Korea and Taiwan (**Figure 5**) [5]. This implied Canada was not testing efficiently to identify active cases, even though testing was being done at a higher rate. In assessing Canada’s testing policies with Taiwan and South Korea, Canada could benefit from less stringent case identification strategy, accuracy, and most importantly, urgency to gain control over the rising number of cases. In the future earlier testing of asymptomatic individuals are needed in Canada in addition to an increased number of testing centres.

### Lessons from the SARS epidemic

The COVID-19 pandemic has greatly surpassed the global impact of the SARS epidemic of 2003 [38]. SARS, also known as SARS-CoV, is a viral respiratory illness that was first reported in February 2003 in Asia and which spread to over 24 countries over a few months, including Canada [38]. SARS and COVID-19 share approximately 79.6% similarity [39].

Although Canada has had experience responding to a similar epidemic (SARS), the country’s response has demonstrated inadequacy in preventing unnecessary hospital visits, research funding and supervision of unreliable media reports. According to the *Learning from SARS* report by David Naylor and other Canada’s top epidemic control experts, about 80% of Dr Naylor’s recommendations were implemented [40,41]. Positive changes that were implemented during COVID-19 include increased communication and collaboration between the federal and provincial governments, construction of negative pressure rooms in hospitals and using PPEs that employees are familiar with [42]. A comparison of the SARS pandemic responses and COVID-19 response is presented in **Table 3** [42]. However, Canada was not able to enact such measures to the needed level according to Naylor, resulting in an overwhelming number of cases being treated in hospitals and a cascade of unnecessary visits [13,41]. Another deficiency was too little funding into biomedical research. Despite recommendations, research and development funding as a whole had decreased since 2001, accounting for only 1.553% of Canada’s total GDP [43]. Compared to other members of the G7 (informal organization with 7 of the world’s most advanced economies), Canada is second last in this regard, with only Italy spending less on research and development [43]. The Scientific Advisory Group assembled by the WHO also outlined urgent research gaps that must be addressed for the resolution of the COVID-19 pandemic [8]. The importance of addressing these gaps is to allow rapid sharing of information so that everyone can have a better understanding of COVID-19, enable policy makers to make optimal decisions, and allow medical professionals to be informed of the newest information. Therefore, Canada should focus on allocating more funding for biomedical research as it is crucial in controlling the pandemic and should aim to become a leading member of research internationally.

**Table 3:** Differences between 2003 and 2020 in preparedness for a novel coronavirus at a large academic hospital (Sunnybrook Hospital) in Toronto, Canada*

	2003: SARS in Toronto	2020: COVID-19 Toronto case
Public health structures and infrastructures:		
Adequate funding and human resources	No	Yes
Protocols for information sharing among different levels of government	No	Yes
Link between public health and hospitals	Weak, fragmented, uncoordinated	Coordination and information sharing present
Rapid and accurate diagnostic testing:	No	Yes
IPAC program structure and related hospital program:		
ICP staffing level	Understaffed: 3 ICPs for 1257 total beds (0.23 ICP/100 beds)	Adequate: 13 ICPs for 1355 total beds (0.96 ICP/100 beds)
ICP certification (Certification Board of Infection Control and Epidemiology)	Not universal	Required
Occupational Health & Safety	Disconnected from IPAC	Coordinated with IPAC
IPAC administrative controls:		
Syndromic triage in ED	No	Yes
Febrile respiratory illness surveillance	No	Yes
Isolation of all patients with acute respiratory symptoms	No	Yes
Awareness of super-spreading events and individuals	No	Yes
Minimizing AGMP and protected intubation policies	No	Yes
Hand hygiene program:	No	Yes
Healthy Workplace Policy (work restrictions for HCWs with acute infectious symptoms)	No	Yes
Presence of a pandemic plan	No	Yes
Engineering and environmental controls:		
Number of airborne infection isolation room	20 (0 in ED)	46 (8 in ED)
ED infrastructure	Shared air system; no protective barrier at triage	Isolated air system with negative pressure in each zone; protective barrier at triage
Terminal disinfection completed twice at patient discharge for high-consequence pathogen	No	Yes
Personal protective Equipment (PPE):		
Regular N95 fit-testing of HCWs	No	Yes
Clear recommendation on PPE for any novel high-consequence pathogen	No	Yes

IPAC − infection prevention and control, ICP − infection prevention and control professional, AGMP − aerosol-generating medical procedure, HWC − healthcare worker, ED − emergency department.

*Adapted from [42].

Lastly, public reaction towards the SARS epidemic underscores the importance of preventing unnecessary fear and panic. In fact, Toronto and its Southeast Asian communities were the subjects of sensational and vastly misleading media articles that aimed to grab its readers’ attention. Asian businesses suffered and the overdramatized media headlines drew attention from important health information [44]. The FBI warned racist hate crimes were on the rise and yet horrific crimes such as the stabbing of an Asian family that included two children under the age of 6 occurred due to fear of contracting the virus [45]. It is essential to educate the public in regards to the stigma towards East Asians through campaigns and support groups. If used effectively, the media would otherwise be extremely useful for medical professionals to rapidly distribute key information and positivity. By preventing the dissemination of false information, it is possible to mitigate the hysteria that usually accompanies these events. Other examples included panic buying of face masks and hand sanitizers that resulted in a shortage for health care providers. Although a mass hysteria caused by the media experienced during the SARS epidemic was avoided, similar reactions of fear and anxiety are observed now. Hence, the social repercussions are a reminder for policymakers to create preventative measures for the emotional consequences of a pandemic.

### Mental health

Mental health is an emotional consequence of priority and concern during the COVID-19 pandemic in multiple countries. A research team at Chiang Mai University found that 44 of 80 suicide attempts in April in Thailand were related to the impacts of the economic challenges of the pandemic [46]. In Canada, a national survey of 1002 participants conducted by the Centre for Addiction and Mental Health showed 21.50% experienced moderate to severe anxiety, 21.20% felt depressed, and 24.70% engaged in binge drinking [47]. Statistics Canada also reported 88% of individuals experienced at least one symptom of anxiety [48]. Anxiety was associated with financial stress, employment impacts and employment exposure to the virus. Asians were also less likely to receive mental health care due to perceived discrimination, which creates more mental distress to Asians suffering from mental health crisis as a result of racism during the pandemic [49,50]. The Asian Pacific Policy and Planning Council & Chinese for Affirmative Action reported that in the United States alone in the first two weeks since March 19, 2020 there were 1135 cases of discrimination and harassment against Asian Americans [51]. This is due to the notion that those of the Asian race may have the coronavirus. Moreover, a mental health gap is established between Asians and other races as a result of discrimination during COVID-19 [49,50]. Asians are also less likely to receive mental health care due to the negative stigma towards mental health in Asian communities [52], which creates more mental distress to Asians suffering from mental health crisis as a result of racism during the pandemic.

In rapid article reviews and clinical studies, low psychological well-being and/or increased symptoms of anxiety, depression, and poor sleep quality were associated with students, health care workers, the female gender, poor self-rating of personal health, COVID-19 patients and patient relatives [53-55]. Two of the reviewed studies reported as high as 96.2% of 714 patients (hospitalized but stable) presented posttraumatic syndrome symptoms (PTSS). This included one descriptive study on alleviating anxiety and depression levels that found pandemic information and preventative health measures, such as proper hand hygiene, could have an effect [53]. Statistical results from reviewed studies varied greatly, but the overall trends remained consistent. On another note, a positive correlation was seen between longer times spent in quarantine and negative impacts on mental health in Italy [56]. This confirmed the importance of minimizing pandemic impacts and in turn, quarantine times.

Most efforts of mental health care took the form of online or over-the-phone services that provide professional support to combat stress, anxiety, and loneliness. Some efforts to lessen the mental impacts of the pandemic were seen through releases by governments and the World Health Organization to reduce the stigma surrounding those being treated for COVID-19 and resources to cope with feelings such as stress, anxiety, and isolation during the pandemic [57]. There have also been efforts through releases by prominent figures and institutions to try and combat targeted racism as a result of COVID-19 [58]. However, there was insufficient data measuring the success of these initiatives thus researchers also recommended testing such initiatives to evaluate their success. Current evidence calls for a great need for evidence-based research on the development of mental health care initiatives as well as preventative and treatment measures. It also called for a need for further research on the effect public communications sent out by governments and organizations had on the mental health of those in quarantine. Research on these topics will help institutions be more prepared in the event of another public health crisis in the future.

### School closures

Studies are inconclusive on whether closing schools helped control the pandemic. There was compelling evidence that school closure reduced transmission of influenza, but it was most effective under conditions of low transmission and higher rate of infection among children [59]. In contrast, modelling studies showed that school closures alone reduced just 2%-4% of total deaths [59]. Transmission between school children was also low in the classroom [60]. For COVID-19, young children below the age of 10 only account for 1% of symptomatic cases [59]. Although no official data can be found on school closures during COVID-19, Taiwan is praised for its success in controlling the COVID-19 pandemic and was one of few regions that promoted local temporary class closures and avoided mass school closures [61]. More data and quality research are required on the effectiveness of widespread school closures. Additionally, the long-term academic and mental health impacts of online school are unknown. Government officials should look to modified interventions in schools to reduce social disruptions and financial burdens.

## CONCLUSION

Canada’s response to COVID-19 was lacking in several vital areas which was evidenced by its negligence of LTC homes, lack of a robust testing strategy, insufficient medical resources, ineffective communications with the public, and underfunding of biomedical research. As a consequence of these deficiencies, combined with the nation’s delayed pandemic measures to protect the health of the public, Canada may have missed a crucial opportunity to contain the spread of the virus in its early stages. In addition, the mental health of health care workers and vulnerable individuals has been labeled a concern and further investigation on mental health interventions is needed. As this study puts forward evidence that the Canadian government was not fully prepared for this pandemic, we proposed areas of further research on early response plans and their effects for Canada. This included but was not limited to the effectiveness of mass school closures and international border closures on reducing viral transmission, region-specific pandemic controls, as well as the importance of timing for testing and case tracing. Early responses are also a very cost-efficient and time-efficient way to take control of a pandemic. To avoid unnecessary and exacerbated outcomes, early response is an important topic of research.

This research, however, is subject to several limitations. First, there was a limited amount of previous research in the field. Fortunately, most of the scientific articles are made open access for rapid research. Second, the findings may not be entirely applicable to other countries as it is tailored to Canada. Third, as this paper was written in the midst of the COVID-19 pandemic, information regarding active cases, mortality rates and testing rates are based on data that is continuously being updated. This puts in question the reliability of certain sources and their ability to present accurate data and statistics as the pandemic moves forward. Additionally, the origin of information used by each source to present data must be dependable. Known credible sources were used to support our claims, however, not all sources were peer-reviewed. Lastly, no ethical issues were present as this study was entirely based on published data. This paper should act as a starting point for more comprehensive and academic research on COVID-19 responses.
